# Ocular manifestations following COVID-19 vaccination

**DOI:** 10.1186/s12348-023-00358-x

**Published:** 2023-09-23

**Authors:** Padmamalini Mahendradas, Sai Bhakti Mishra, Rohini Sangoram, Sanjay Srinivasan, Ankush Kawali, Aditya Patil, Rohit Shetty

**Affiliations:** 1 Department of Uveitis and Ocular Immunology, Narayana Nethralaya, Bangalore, India; 2 Department of Cornea and Refractive Surgery, Narayana Nethralaya, Bangalore, India

**Keywords:** COVID-19, SARS-CoV-2, Vaccination, Uveitis, Scleritis, Ocular inflammation, Immunologic, Coronavirus disease, Vaccine-associated uveitis

## Abstract

**Background:**

Immunologic and inflammatory adverse effects following vaccination against COVID-19 are being reported. While some reactions may develop denovo others concern its immunogenic effect in patients with pre-existing inflammatory conditions.

**Methods:**

Retrospective consecutive patients diagnosed with ocular inflammatory manifestations within 8 weeks of receiving COVID-19 vaccination who presented to a tertiary eye care centre in South India.

**Results:**

Ninety-eight eyes of 67 patients presenting with ocular inflammatory manifestations within 8 weeks following COVID-19 vaccination were studied. The mean age was 43 years (+/- 14.82; range 19–80 years). The most common presentations were anterior uveitis (*n* = 31, 31.7%), followed by panuveitis (*n* = 24, 24.5%). The mean time to onset of symptoms was 25 days (+/- 15.48; range 2–55 days) following a dose of vaccine.

Among all patients, 39 (58.2%) had a previous history of ocular inflammation. Mean presenting visual acuity was 0.4 (0-4) logMAR units and mean final visual acuity was 0.2 (0-4) logMAR units. The causes for reduced vision included of cystoid macular edema (*n*=2, 2%), chorioretinal atrophy (*n*=2.2%), optic atrophy (*n*=1.1%), retinal vascular occlusion (*n*=1.1%) and acute retinal necrosis (*n*=1.1%).

**Conclusion:**

Infective and immunogenic adverse events should be watched out for after COVID-19 vaccination. It is difficult to establish causality for such manifestations, nevertheless, most of them were mild and had good final visual outcomes.

**Supplementary Information:**

The online version contains supplementary material available at 10.1186/s12348-023-00358-x.

## Introduction

Anti-SARS CoV-2 (Severe acute respiratory syndrome coronavirus 2) vaccination has played a vital role in the control of the COVID-19 (Coronavirus disease 2019) pandemic. As of 26 February 2022, a total of 10,585,766,316 vaccine doses have been administered across the world [1]. Similar to previous several other vaccines, COVID-19 vaccinations have not been without adverse effects, most of which have been reported largely from post-marketing surveillance phases [2]. Hence it is critical to report any adverse effects occurring after vaccination for improving our understanding of not only de novo reactions but also its effects on previously existing illness such as autoimmune as well as infectious diseases.

Various vaccine models have been approved and each type of vaccine for COVID-19 works differently to introduce antigens, which are unique features of the SARS-CoV-2 virus, into the human body. These antigens trigger a specific immune response which ultimately is responsible for building immune memory and helps the body counter SARS-CoV-2 infections in the future. Currently, four types of COVID- 19 vaccines are available, including the vector vaccines (Janssen Johnson & Johnson and Oxford-AstraZeneca, COVISHIELD™, SputnikV); the whole virus vaccines (Sinovac13, Sinopharm14 and Covaxin); the messenger RNA (mRNA) vaccines (Pfizer-BioNTech and Moderna); and the protein subunit vaccines (Novavax, corbevax). Covovax SARS-CoV-2 rS Protein (COVID-19) recombinant spike protein Nanoparticle Vaccine. Inflammatory adverse events, including pericarditis, myocarditis and multisystem inflammatory syndrome, have been reported to occur following COVID-19 vaccination [3–5]. The objective of this study is to describe the various ocular inflammatory manifestations associated with the administration of COVID-19 vaccination in an Indian population.

## Methods

Retrospective case series of patients diagnosed with ocular inflammatory manifestations after receiving COVID-19 vaccination were collected from hospital medical records in the uveitis and ocular immunology department in a tertiary care referral eye hospital in South India over a 3-month period in 2021. The study was conducted with institutional ethical committee approval. A diagnosis of COVID-19 vaccination triggered ocular inflammation was established based on the onset of the symptoms within 8 weeks following COVID-19 vaccination.

The following data were retrieved from patients’ medical records: age, gender, laterality, type of COVID-19 vaccination, doses of vaccination, time to onset of symptoms, past ocular history, type of ocular inflammatory manifestation (anatomical diagnosis) including anterior uveitis, intermediate uveitis, posterior uveitis, panuveitis, episcleritis, scleritis, sclerouveitis, sclerokeratouveitis, and keratouveitis, etiological diagnosis, treatment and outcome. Statistical data were analyzed using Excel and SSPS statistical program. Continuous variables were described as mean [range] while binary variables were described as numbers (%). A minimum follow-up for a period of 3 months was recorded in all patients. The study and data collection were conducted following the approval by the Institutional review board and ethics committee.

## Results

Ninety-eight eyes of 67 patients presented with ocular inflammatory manifestations within 8 weeks following COVID-19 vaccination. Demographic distribution and vaccination data are described with details of ocular manifestations with anatomical diagnosis in Table 1. The mean age of presenting population was 43 years (+/- 14.82; range 19–80 years) with 53.7% males and 46.3% females. Bilateral involvement was noted in 31 (46.3%) cases. In unilateral cases, right eye was involved in 21 (58.3%) cases.

**Table 1:** Table providing information about all patients, type of vaccines, ocular manifestations

Case no.	Age	Gender	Vaccine	Dose	Days	Eye	Anatomical diagnosis	Etiological diagnosis	Occurance	Presenting va	Final va
1	72	M	Covishield	1st	12	RE	PU	VIRAL-VZV	FIRST	1.0	0.6
2	42	M	Covishield	1st	45	BE	ES	VIRAL-HSV	FIRST	0.0	0.0
3	62	F	Covishield	1st	15	RE	AU	IDIO	FIRST	0.0	0.0
4	55	M	Covishield	2nd	26	LE	PU	IDIO	FIRST	0.0	0.0
5	32	M	Covishield	1st	16	RE	AU	HLA B27	RECURRENCE	0.0	0.0
6	38	M	Covaxin	2nd	2	BE	PANU	SARCOID	RECURRENCE	0.0	0.0
7	56	M	Covaxin	2nd	25	RE	AU	IDIO	FIRST	0.0	0.0
8	42	M	Covaxin	2nd	18	RE	AU	HLA B27	RECURRENCE	0.0	0.0
9	33	M	Covishield	1st	16	RE	PU	TB	FIRST	0.0	0.0
10	80	M	Covishield	2nd	12	BE	AU	IDIO	RECURRENCE	0.2	0.0
11	26	M	Covishield	1st	40	BE	PANU	VKH	FIRST	1.0	0.9
12	67	F	Covishield	2nd	35	LE	KU	VIRAL-VZV	RECURRENCE	0.6	0.4
13	26	F	Covishield	2nd	30	RE	AU	IDIO	FIRST	0.0	0.0
14	34	M	Covishield	1st	7	RE	AU	IDIO	FIRST	0.1	0.0
15	28	M	Covishield	2nd	13	RE	ES	IDIO	FIRST	0.0	0.0
16	58	F	Covishield	1st	10	BE	PANU	VKH	FIRST	0.5	0.2
17	36	M	Covishield	2nd	30	RE	KU	IDIO	FIRST	0.2	0.0
18	55	F	Covaxin	1st	15	RE	AU	VIRAL-VZV	RECURRENCE	1.2	0.7
19	67	F	Covishield	1st	5	RE	SU	VIRAL-VZV	RECURRENCE	2.0	3.0
20	77	M	Covishield	1st	30	LE	KU	VIRAL-UNCLASSIFIED	FIRST	0.7	0.3
21	25	M	Covishield	1st	5	BE	AU	IDIO	RECURRENCE	0.2	0.0
22	51	F	Covishield	1st	40	BE	PANU	SARCOID	RECURRENCE	0.0	0.0
23	37	M	Covishield	1st	16	BE	PANU	VKH	RECURRENCE	0.5	0.0
24	50	M	Covishield	2nd	10	RE	AU	IDIO	RECURRENCE	0.0	0.0
25	42	M	Covishield	1st	45	BE	ES	VIRAL-UNCLASSIFIED	FIRST	0.0	0.0
26	26	F	Covishield	2nd	15	BE	SKU	HLA B27	RECURRENCE	0.0	0.0
27	46	F	Covishield	1st	11	BE	ES	IDIO	RECURRENCE	0.3	0.0
28	55	F	Covishield	1st	45	RE	AU	HLA B27	RECURRENCE	0.0	0.0
29	55	M	Covishield	1st	30	LE	AU	VIRAL-VZV	FIRST	0.2	0.0
30	74	M	Covishield	2nd	20	BE	AU	VIRAL-VZV	RECURRENCE	0.5	0.0
31	42	M	Covishield	1st	45	BE	ES	VIRAL-UNCLASSIFIED	FIRST	0.0	0.0
32	37	M	Covaxin	2nd	2	BE	PANU	SARCOID	RECURRENCE	0.0	0.0
33	47	M	Covaxin	2nd	18	RE	AU	HLA B27	RECURRENCE	0.0	0.0
34	31	F	Covishield	2nd	50	RE	SU	TB	RECURRENCE	0.0	0.0
35	37	M	Covishield	1st	22	BE	PANU	SARCOID	RECURRENCE	0.0	0.0
36	46	M	Covishield	1st	30	LE	AU	HLA B27	RECURRENCE	0.0	0.0
37	53	F	Covishield	2nd	28	BE	IU	IDIO	RECURRENCE	0.2	0.0
38	60	M	Covishield	2nd	2	RE	KU	VIRAL-UNCLASSIFIED	RECURRENCE	3.0	0.0
39	39	F	Covishield	1st	15	BE	IU	VIRAL-UNCLASSIFIED	RECURRENCE	2.0	1.3
40	37	F	Covishield	1st	30	LE	AU	HLA B27	RECURRENCE	0.2	0.0
41	46	F	Covishield	2nd	55	RE	AU	HLA B27	RECURRENCE	0.2	0.0
42	32	M	Covishield	1st	16	RE	AU	HLA B27	RECURRENCE	0.2	0.0
43	19	M	Covishield	2nd	21	RE	AU	IDIO	RECURRENCE	0.0	0.0
44	52	M	Covishield	1st	13	LE	AU	HLA B27	RECURRENCE	0.2	0.0
45	47	M	Covishield	1st	7	BE	PU	IDIO	FIRST	0.8	0.2
46	56	F	Covishield	1st	45	LE	AU	VIRAL-VZV	FIRST	0.0	0.0
47	19	F	Covishield	2nd	25	BE	IU	IDIO	FIRST	0.0	0.0
48	35	F	Covishield	1st	55	LE	AU	HLA B27	RECURRENCE	0.2	0.0
49	49	F	Covishield	2nd	55	BE	PANU	IDIO	FIRST	3.0	3.0
50	34	F	Covishield	2nd	50	BE	IU	TB	FIRST	0.0	0.0
51	35	F	Covishield	2nd	45	RE	AU	IDIO	RECURRENCE	0.0	0.0
52	23	F	Covishield	2nd	50	BE	AU	IDIO	RECURRENCE	0.2	0.0
53	49	F	Covishield	2nd	30	BE	PU	IDIO	FIRST	0.2	0.0
54	28	M	Covishield	1st	50	LE	PANU	IDIO	FIRST	0.0	0.0
55	26	M	Covishield	1st	30	BE	SU	HLA B27	RECURRENCE	0.0	0.0
56	47	F	Covaxin	1st	15	LE	ES	IDIO	RECURRENCE	4.0	4.0
57	35	M	Covishield	1st	30	BE	PANU	SARCOID	RECURRENCE	0.0	0.0
58	55	M	Covishield	2nd	55	LE	AU	IDIO	RECURRENCE	0.2	0.0
59	52	F	Covishield	1st	40	LE	S	IDIO	RECURRENCE	0.0	0.0
60	48	F	Covaxin	1st	30	LE	PANU	SARCOID	RECURRENCE	0.2	0.0
61	40	F	Covishield	1st	30	BE	AU	VIRAL-HSV	FIRST	0.0	0.0
62	22	M	Covishield	2nd	10	BE	PANU	VKH	FIRST	0.3	0.2
63	19	F	Covishield	1st	15	BE	PU	IDIO	FIRST	1.2	0.3
64	29	M	Covishield	2nd	30	BE	PANU	IDIO	FIRST	0.0	0.0
65	25	F	Pfizer	Both	3	BE	PU	IDIO	RECURRENCE	0.0	0.0
66	52	F	Sputnik	1st	5	LE	PU	IDIO	FIRST	0.6	0.6
67	19	F	Covaxin	Both	22	BE	IU	JIA	RECURRENCE	0.6	0.2

A large majority of patients, 56 cases (83.6%), had received one or more doses of COVISHIELD™ vaccine. Among the remaining 11 patients, 9 had received Covaxin, while Sputnik and Pfizer vaccines had been administered to one patient each. The mean time to onset of symptoms following vaccination was 25 days (+/- 15.48; range 2–55 days). Ocular inflammation was reported after the first dose of vaccination in 31 (55.2%) cases and after the second dose in 28 (41.8%) cases. Only 2 (3%) patients had developed symptoms after both doses of vaccination.

Anterior uveitis occurred following vaccination in 31 eyes of 26 patients (31.7%). Intermediate uveitis was noted in 10 eyes of 5 patients (10.2%). Twelve eyes of 8 patients (12.2%) had posterior uveitis including occlusive retinal vasculitis. Panuveitis occurred in 24 eyes of 13 patients (24.5%). Episcleritis, scleritis, keratouveitis and sclerokeratouveitis were seen in 10, 1, 4 and 4 eyes respectively. Clinical and laboratory data revealed infectious etiology in 17 (25.4%) patients, while an autoimmune disease was found in 23 (34.3%) patients. Twenty-seven (40.3%) patients were found to have an idiopathic inflammation on routine investigations.

Infectious etiology was predominantly noted to be of viral etiology. Viral uveitis consisted of 14 cases (20.8%). Varicella zoster reactivation was seen in 7 cases (50%), herpes simplex was noted in 2 cases (14%) and 5 cases (36%) had an unclassified viral etiology. Tuberculosis was detected in 3 cases (21.4%). Autoimmune diseases identified were HLA B27 associated uveitis in 12 cases (52.2%), sarcoidosis in 6 cases (26%), Vogt Koyanagi Harada disease occurred in 4 cases (17.8%) and one patient of juvenile idiopathic arthritis had a recurrence of previously quiescent inflammation following vaccination for COVID-19.

De novo inflammation was noted in 28 (41.7%) patients while 39 (58.2%) patients with a formerly diagnosed uveitis, had a recurrence of previously quiescent inflammation post vaccination. Among patients with first episode of ocular inflammation following COVID-19 (28 cases, 41.7%), infectious etiology comprised 10 cases (35%) cases of which viral disease was noted in 8 cases (80%) and tuberculosis in 2 cases (20%). First episode of autoimmune disease associated ocular inflammation was seen in only 3 cases of Vogt Koyanagi Harada disease. Among patients with recurrence of previously quiescent inflammation (39 cases, 58.2%), autoimmune disease association was noted in 20 cases (51.3%). HLA B27 associated disease was noted in 12 cases (60%), sarcoidosis in 6 cases (30%), JIA-related uveitis and VKH disease in 1 case each. Recurrence of old viral uveitis was seen in 6 cases (22.2%) and one case of recurrence of tubercular uveitis was seen.

Mean presenting visual acuity was 0.4 (0–4) logMAR units and mean final visual acuity was 0.2 (0–4) logMAR units. The causes for reduced vision included of cystoid macular edema, chorioretinal atrophy, optic atrophy, retinal vascular occlusion, retinoschisis, retinal detachment and acute retinal necrosis.

## Discussion

Vaccines were manufactured to protect from SARS-CoV-2, the virus causing COVID-19. While vaccines can induce large quantities of high affinity virus-neutralizing antibodies to prevent infection, they haven’t been able to avoid untoward side effects. Vaccination trials required detailed clinical management, complemented with precise evaluation of immune responses and safety. Since the introduction of COVID-19 vaccinations, there have been numerous reports on the adverse ocular events following vaccination Here, we reviewed the ocular inflammatory manifestations of different vaccines in patients who presented with de novo and recurrent ocular inflammation following vaccination against SARS-CoV-2.

Possible pathological mechanisms responsible for autoimmune responses in SARS-CoV-2 infected patients include molecular mimicry, bystander activation, epitope spreading, cryptic antigen presentation, B-cell polyclonal activation and the existence of the superantigens have been suggested as possible pathological mechanisms behind autoimmune responses in SARS-CoV-2 infected patients [6, 7]. Either of these mechanisms might have resulted in recurrence of inflammation in our cases.

Uveitis and other ocular inflammatory events have been described following vaccinations for Bacille-Calmette- Guerin (BCG), hepatitis B virus (HBV), hepatitis A virus (HAV), varicella virus, human papillomavirus (HPV), influenza virus, measles-mumps-rubella (MMR), yellow fever, and typhoid [8–15]. Furthermore, various ocular adverse events have also been reported after COVID-19 vaccination.

Although the precise pathogenesis frequently remains unclear, different mechanisms have been hypothesized including changes in adaptive and innate immune system. Both arms of the immune system may be influenced by adjuvants via various mechanisms including the activation of.

Toll-like receptors, Nucleotide-binding and oligomerization domain (NOD)-like receptors (NLRs) etc., leading to downstream in cytokines generation. Moreover, the heightened adaptive immune response to antigen can occur following the activation of antigen presenting cells [16–18]. Other mechanisms include the direct infection by the attenuated but active virus strain and inflammation induced by one or more adjuvants (such as aluminum salts), routinely used in inactivated or subunit/conjugate vaccines [16, 19]. Patients with a personal or history of autoimmune disease may develop auto-inflammation or autoimmune conditions induced by adjuvants, known as Shoenfeld syndrome. [16, 20].

Testi I et al. reported a multinational case series of 70 patients diagnosed with inflammatory adverse events following COVID-19 vaccination from 40 centres over a 3-month period [21]. They found the mean age as 51 years (range 19–84 years). Most common events noted were anterior uveitis (58.6%) followed by posterior uveitis 12.9% and the mean time to adverse event was 5 days after the first vaccine and 6 days following the second dose of vaccine.

Rabinovitch et al. described 21 cases of uveitis following the administration of the BNT162b2 mRNA vaccine in Israel, of which nineteen (90.5%) were diagnosed with anterior uveitis [22]. Pichi et al. reported seven patients diagnosed with acute macular neuroretinopathy (AMN) (2), paracentral acute middle maculopathy (PAMM) (1) subretinal fluid (1), episcleritis (1), and anterior scleritis (2) following soon after receiving a dose of inactivated COVID-19 vaccination (Sinopharm) [23].

Bolletta E et al. reported 34 patients with uveitis and other ocular complications following COVID-19 vaccination. With a mean age of 49.8 years (range 18–83 years). Mean time between vaccination and the onset of ocular complication was 9.4 days (range 1–30 days) [24]. Pang et al. reported 12 eyes of 9 cases of ocular adverse events following administration of inactivated COVID-19 vaccines, although the causal relationship could not be established in the study [25]. The mean (SD) age was 44.7 ± 16.5 years (range, 19– 78 years), with 77.8% female cases. The mean time of ocular adverse events was 7.1 days (range, 1–14 days) after receiving the inactivated COVID-19 vaccine. They described patients with choroiditis, optic disc vasculitis with sunset glow fundus, keratitis, scleritis, acute retinal necrosis and anterior uveitis [25].

Studies have reported ocular inflammation following most of the vaccines administered worldwide including Pfizer-BioNTech vaccination (BNT162b2 mRNA), Oxford-AstraZeneca vaccine (ChAdOx1 nCoV-19) which is similar to Covishield available in India, ModernaTX vaccination (mRNA-1273), and Janssen Johnson & Johnson vaccine (Ad26.COV2) [25]. It is difficult comment on the comparative immunogenicity and safety of different vaccines due to the geographical distribution bias of vaccinations administered. Nevertheless, systemic and local side-effects after BNT162b2 and ChAdOx1 nCoV-19 vaccination that were studied prospectively using an app in the UK showed that the frequencies were lower than reported in phase 3 trials [26].

Episcleritis and scleritis have also been reported in patients within a mean of 5 days following the first dose of the inactivated COVID-19 vaccine (Sinopharm) [24, 25]. Renisi et al. reported a case of anterior uveitis 14 days after the second dose of Pfizer–BioNTech COVID-19 vaccine [27]. One case of a 18-year-old female with a history of antinuclear antibody (ANA) positive oligoarticular juvenile idiopathic arthritis (JIA) developing bilateral anterior uveitis 5 days after the second dose of BBIBP-CorV has been reported [28]. We also had a 19-year old patient with previously quiescent inflammation with JIA associated uveitis that recurred following both doses of vaccination. Increased IFN-I secretion by vaccine-induced immunological responses could potentially generate autoimmune manifestations in patients with a history of systemic autoimmunity disease has been the proposed hypothesis in such cases [24, 28, 29].

Vaccines can result in the reactivation of varicella zoster virus (VZV), has been previously described in patients receiving vaccines for rabies, hepatitis A, influenza, and Japanese encephalitis [30]. There are cases reported in the literature describing VZV reactivation after vaccination with the mRNA COVID-19 vaccine, including cases with herpes zoster ophthalmicus (HZO) [31–34]. We have reported a case of acute retinal necrosis in a 71 year male following COVISHIELD vaccination due to VZV reactivation [35].

Although unclear, the proposed hypothesis of stimulation of the immune system that induces a strong T-cell response following vaccination. This can lead to an increased CD8 + T cell and T helper type 1 CD4 + T cell population with relatively low VZV-specific CD8 + cells, that further allows VZV to escape its latent phase. Abrogation in TLR expression among vaccinated individuals is another possible explanation that has been linked with marked induction of type I interferon (IFN-I) and potentiation of pro-inflammatory cytokines. This may negatively modulate antigen expression and potentially contribute to VZV reactivation [33]. Bolletta E et al. reported two cases of herpes keratitis reactivation in patients with a history of previous herpetic keratouveitis although under systemic antiviral treatment [24]. There has also been a speculation regarding the preventive role of antiviral treatment in patients with prior history of herpes [34].

Recurrence of Toxoplasma retinochoroiditis have been described due to vaccination-induced CD8 T-cell exhaustion that may lead to parasite reactivation [25, 36], however, we did not have any cases of toxoplasmosis in our study. Arora A et al. described two patients with recurrence of tubercular choroiditis 2–6 weeks following the first dose of Covishield vaccine. As both their patients had unilateral recurrence, they were managed with intravitreal injection dexamethasone (0.7 mg) (Ozurdex®, Allergan, Inc., Irvine, CA, USA) [37]. We had three patients with tubercular uveitis, one each with de novo intermediate uveitis, de novo serpiginous like choroiditis and recurrence of sclerouveitis.

We found a majority of HLA B27 related uveitis (60%) among the non-infective uveitis cases in our study. All 100% of these patients had developed a recurrence of their previous quiescent inflammation. There have been similar reports of vaccine triggered inflammation in HLA B27 positive cases by other authors [21, 24]. Furer V et al. reported mRNA BNTb262 vaccine as immunogenic in majority of patients with autoimmune inflammatory rheumatic diseases. Factors responsible for reduced immunogenicity included those on treatment with glucocorticoids, rituximab, MMF, and abatacept [38].

Other autoimmune pathologies included reactivation of inflammation in 6 patients with sarcoidosis and one patient with VKH. However, there were 3 patients with first episode of VKH within the study period. Dysregulation of the immune system and other immunological mechanisms may play a significant role in the association between VKH disease and COVID-19 vaccination [39–41].

Papasavvas I. and Herbort CP. also have reported a case of VKH disease which was completely under control for 6 years and on infliximab maintenance therapy that was reactivated 6 weeks after the second dose of the Pfizer vaccine administration [42]. Saraceno JJF et described a case of a 62-year-old healthy female who developed complete Vogt-Koyanagi-Harada (VKH) Syndrome 4 days after a dose of ChAdOx1 nCoV-19 (AZD1222) vaccine [43]. There have also been reports of exaggeration of VKH disease following Covid-19 vaccination where the close temporal relationship between the vaccine dose and the worsening of symptoms strongly suggested COVID-19 vaccination as the trigger of its exacerbation [44].

Concerning post-vaccination thrombosis, there are reports of central retinal vein occlusion, branch retinal vein occlusions and superior ophthalmic vein thrombosis [24, 45–48]. In addition to major blood vessel occlusions, there are also reports of capillary plexus occlusions including cases with AMN following Covid-19 vaccination [49–52]. Similarly, Bohler AD et al. reported a case of AMN in a young woman who was taking combined estrogen–progestin oral contraceptives 2 days after ChAdOx1 nCoV-19 vaccination [44]. We also report here a 25-year-old female patient who developed bilateral sequential AMN following both doses of vaccine [53].

A recently reported retrospective study assessed the risk of vaccine associated uveitis (VAU) following SARS-CoV-2 vaccination in 1094 cases from 40 countries [54]. They found most cases were reported in patients who received Pfizer-BioNTech vaccine. The mean age of patients with VAU was 46.24 ± 16.93 years, and majority were women (68.65%) Most cases were reported after the first dose (41.32%) and within the first week (54.02%) of the vaccination.

The major limitation of this study was its retrospective design. Other limitations include a relatively low number of cases, single tertiary referral centre bias and a limited period study. There is growing evidence in literature about the ocular complications following COVID- 19 vaccination, although a definitive association has been difficult to demonstrate.

Inflammatory ocular manifestation in the anterior and posterior segments may be seen in following COVID-19 vaccine. Considering the growing number of COVID-19 vaccinations and the commencement of booster vaccines, increasing number of ocular inflammatory adverse effects may be seen from the various available vaccines. Even if causality remains presumed, caution must be warranted among physicians about the possibility of ocular inflammation following SARS-CoV-2 vaccination.

## Conclusion

Improved reporting and enhanced understanding of mechanisms responsible for post-vaccination ocular inflammation can help in well-designed vaccination strategies. These are critical for establishing better guidelines and providing insights to limit harm while promoting assurance especially when a large population including pediatric age-group are yet to receive their vaccines. Nevertheless, we still consider vaccination as a vital public health tool in the containment of the COVID-19 pandemic.

### Supplementary Information

**Additional file 1: Graph 1.** Percentage of population affected (in weeks) following a dose of COVID-19 vaccination. **Graph 2.** Type of occurrence of inflammatory manifestations following dose of vaccine. **Graph 3.** Etiological diagnosis and type of occurrence showing that cases with autoimmune uveitis primarily showed recurrence of inflammation and rarely presented with a first episode of disease

## Data Availability

Available on requests.

## Abbreviations

COVID-19: Coronavirus disease 19
SARS Cov-2: Severe acute respiratory syndrome coronavirus 2
JIA: Juvenile idiopathic arthritis
VKH: Vogt-Koyanagi-Harada
HLA B27: Human leucocyte antigen B27
ANA: Anti-nuclear antibodies
VZV: Varicella-zoster virus

## References

[1] World Health Organization. WHO Coronavirus (COVID-19) Dashboard. Available at https://covid19.who.int/ [Accessed 26 Feb 2022]

[2] Kaur RJ, Dutta S, Bhardwaj P, Charan J, Dhingra S, Mitra P, Singh K, Yadav D, Sharma P, Misra S (2021). Adverse events reported from COVID-19 vaccine trials: a systematic review. Indian J Clin biochemistry: IJCB.

[3] Montgomery J, Ryan M, Engler R, Hoffman D, McClenathan B, Collins L, Loran D, Hrncir D, Herring K, Platzer M, Adams N, Sanou A, Cooper LT (2021). Myocarditis following immunization with mRNA COVID-19 vaccines in members of the US military. JAMA Cardiol.

[4] Pepe S, Gregory AT, Denniss AR (2021). Myocarditis, Pericarditis and Cardiomyopathy after COVID-19 vaccination. Heart Lung Circ.

[5] Park JW, Yu SN, Chang SH, Ahn YH, Jeon MH (2021). Multisystem inflammatory syndrome in an adult after COVID-19 vaccination: a Case Report and Literature Review. J Korean Med Sci.

[6] Caso F, Costa L, Ruscitti P, Navarini L, Del Puente A, Giacomelli R, Scarpa R (2020). Could Sars-coronavirus-2 trigger autoimmune and/or autoinflammatory mechanisms in genetically predisposed subjects?. Autoimmun rev.

[7] Ehrenfeld M, Tincani A, Andreoli L, Cattalini M, Greenbaum A, Kanduc D, Alijotas-Reig J, Zinserling V, Semenova N, Amital H, Shoenfeld Y (2020). Covid-19 and autoimmunity. Autoimmun rev.

[8] Fraunfelder FW, Suhler EB, Fraunfelder FT (2010). Hepatitis B vaccine and uveitis: an emerging hypothesis suggested by review of 32 case reports. Cutan Ocul Toxicol.

[9] Holt HD, Hinkle DM, Falk NS, Fraunfelder FT, Fraunfelder FW (2014). Human papilloma virus vaccine associated uveitis. Curr Drug Saf.

[10] Sood AB, O’Keefe G, Bui D, Jain N (2019). Vogt-Koyanagi-Harada disease associated with hepatitis B vaccination. Ocul Immunol Inflamm.

[11] Escott S, Tarabishy AB, Davidorf FH (2013). Multifocal choroiditis following simultaneous hepatitis a, typhoid, and yellow fever vaccination. Clin Ophthalmol.

[12] Abou-Samra A, Tarabishy AB (2019). Multiple evanescent white dot syndrome following intradermal influenza vaccination. Ocul Immunol Inflamm.

[13] Biancardi AL, Moraes HV (2019). Anterior and intermediate uveitis following yellow fever vaccination with fractional dose: case reports. Ocul Immunol Inflamm.

[14] Benage M, Fraunfelder FW (2016). Vaccine-associated uveitis. Mo Med.

[15] Cunningham ET, Moorthy RS, Fraunfelder FW, Zierhut M (2019). Vaccine- associated uveitis. Ocul Immunol Inflamm.

[16] Israeli E, Agmon-Levin N, Blank M, Shoenfeld Y (2009) Adjuvants and autoimmunity. Lupus. 18:1217–1225. 10.1177/0961203309345724 10.1177/0961203309345724 19880572

[17] Coffman RL, Sher A, Seder RA (2010). Vaccine adjuvants: putting innate immunity to work. Immunity.

[18] Watad A, Sharif K, Shoenfeld Y (2017). The ASIA syndrome: basic concepts. Mediterr J Rheumatol.

[19] McElrath MJ (1995). Selection of potent immunological adjuvants for vaccine construction. Semin Cancer Biol.

[20] Watad A, Quaresma M, Brown S, Cohen Tervaert JW, Rodríguez- Pint I, Cervera R et al (2017) Autoimmune/inflammatory syndrome induced by adjuvants (Shoenfeld’s syndrome)—an update. Lupus. 26:675– 81. doi: 10.1177/0961203316686406 10.1177/0961203316686406 28059022

[21] Testi I, Brandão-de-Resende C, Agrawal R, Pavesio C, COVID-19 Vaccination Ocular Inflammatory Events Study Group (2022). Ocular inflammatory events following COVID-19 vaccination: a multinational case series. J ophthalmic Inflamm Infect.

[22] Rabinovitch T, Ben-Arie-Weintrob Y, Hareuveni-Blum T, Shaer B, Vishnevskia- Dai V, Shulman S, Newman H, Biadsy M, Masarwa D, Fischer N, Yovel O, Ben Zaken SG, Habot-Wilner Z (2021). Uveitis following the BNT162b2 mRNA vaccination against SARS-CoV-2 infection: a possible association. Retina 2.

[23] Pichi F, Aljneibi S, Neri P, Hay S, Dackiw C, Ghazi NG (2021). Association of Ocular adverse events with inactivated COVID-19 vaccination in patients in Abu Dhabi. JAMA Ophthalmol.

[24] Bolletta E, Iannetta D, Mastrofilippo V, De Simone L, Gozzi F, Croci S, Bonacini M, Belloni L, Zerbini A, Adani C, Fontana L, Salvarani C, Cimino L (2021). Uveitis and other ocular complications following COVID-19 vaccination. J Clin Med.

[25] Pang K, Pan L, Guo H, Wu X (2022). Case Report: Associated Ocular adverse reactions with inactivated COVID-19 vaccine in China. Front Med (Lausanne).

[26] Menni C, Klaser K, May A, Polidori L, Capdevila J, Louca P, Sudre CH, Nguyen LH, Drew DA, Merino J, Hu C, Selvachandran S, Antonelli M, Murray B, Canas LS, Molteni E, Graham MS, Modat M, Joshi AD, Mangino M, Hammers A, Goodman AL, Chan AT, Wolf J, Steves CJ, Valdes AM, Ourselin S, Spector TD (2021). Vaccine side-effects and SARS-CoV-2 infection after vaccination in users of the COVID Symptom Study app in the UK: a prospective observational study. Lancet Infect Dis.

[27] Renisi G, Lombardi A, Stanzione M, Invernizzi A, Bandera A, Gori A (2021). Anterior uveitis onset after bnt162b2 vaccination: is this just a coincidence?. Int J Infect Dis.

[28] ElSheikh RH, Haseeb A, Eleiwa TK, Elhusseiny AM (2021) Acute uveitis following COVID-19 vaccination. Ocul Immunol Inflamm Aug11 1–3. 10.1080/09273948.2021.1962917 10.1080/09273948.2021.1962917 34379565

[29] Sprent J, King C (2021). COVID-19 vaccine side effects: the positives about feeling bad. Sci Immunol.

[30] Walter R, Hartmann K, Fleisch F, Reinhart WH, Kuhn M (1999). Reactivation of herpesvirus infections after vaccinations?. Lancet.

[31] Eid E, Abdullah L, Kurban M, Abbas O (2021). Herpes zoster emergence following mRNA COVID-19 vaccine. J Med Virol.

[32] Papasavvas I, De Courten C, Herbort CP (2021). Jr. Varicella-zoster virus reactivation causing herpes zoster ophthalmicus (HZO) after SARS-CoV-2 vaccination–report of three cases. J Ophthalmic Inflamm Infect.

[33] Triantafyllidis KK, Giannos P, Mian IT, Kyrtsonis G, Kechagias KS (2021). Varicella zoster virus reactivation following COVID-19 vaccination: a systematic review of case reports. Vaccines.

[34] Herbort CP, Papasavvas I (2021). Effect of SARS-CoV-2 mRNA vaccination on ocular herpes simplex and varicella-zoster virus reactivation: should preventive antiviral treatment be given in known herpes patients. J Ophthalmic Inflamm Infect.

[35] Mishra SB, Mahendradas P, Kawali A, Sanjay S, Shetty R Reactivation of varicella zoster infection presenting as acute retinal necrosis post COVID 19 vaccination in an asian indian male. Eur J Ophthalmol 2021 Sep 18:11206721211046485. doi: 10.1177/11206721211046485 10.1177/11206721211046485 34541931

[36] Khan IA, Ouellette C, Chen K, Moretto M, Toxoplasma (2019). Immunity and pathogenesis. Curr Clin Microbiol Rep.

[37] Arora A, Handa S, Singh SR, Sharma A, Bansal R, Agrawal R, Gupta V (2022 Mar) Recurrence of tubercular choroiditis following anti-SARS-CoV-2 vaccination. Eur J Ophthalmol 21:11206721221088439. 10.1177/11206721221088439Epub ahead of print. PMID: 35306917; PMCID: PMC8938687 10.1177/11206721221088439 PMC8938687 35306917

[38] Furer V, Eviatar T, Zisman D, Peleg H, Paran D, Levartovsky D, Zisapel M, Elalouf O, Kaufman I, Meidan R, Broyde A, Polachek A, Wollman J, Litinsky I, Meridor K, Nochomovitz H, Silberman A, Rosenberg D, Feld J, Haddad A, Gazzit T, Elias M, Higazi N, Kharouf F, Shefer G, Sharon O, Pel S, Nevo S, Elkayam O (2021). Immunogenicity and safety of the BNT162b2 mRNA COVID-19 vaccine in adult patients with autoimmune inflammatory rheumatic diseases and in the general population: a multicentre study. Ann Rheum Dis.

[39] Dogan B, Erol MK, Cengiz A (2016). Vogt-Koyanagi-Harada disease following BCG vaccination and tuberculosis. Springerplus.

[40] Bricout M, Petre A, Amini-Adle M, Bezza W, Seve P, Kodjikian L, Dalle S, Thomas L (2017). Vogt-Koyanagi-Harada-like syndrome complicating pembrolizumab treatment for metastatic melanoma. J Immunother.

[41] Kim M (2016). Vogt-Koyanagi-Harada Syndrome following influenza vaccination. Indian J Ophthalmol.

[42] Papasavvas I, Herbort CP (2021). Reactivation of Vogt-Koyanagi-Harada disease under control for more than 6 years, following anti-SARS-CoV-2 vaccination. J Ophthalmic Inflamm Infect.

[43] Saraceno JJF, Souza GM, Dos Santos Finamor LP, Nascimento HM, Belfort R (2021). Vogt-Koyanagi-Harada Syndrome following COVID-19 and ChAdOx1 nCoV-19 (AZD1222) vaccine. Int J Retina Vitreous.

[44] De Domingo B, López M, Lopez-Valladares M, Ortegon-Aguilar E, Sopeña-Perez-Argüelles B, Gonzalez F (2022). Vogt-Koyanagi-Harada Disease Exacerbation Associated with COVID-19 vaccine. Cells.

[45] Bayas A, Menacher M, Christ M, Behrens L, Rank A, Naumann M (2021). Bilateral superior ophthalmic vein thrombosis, ischaemic stroke, and immune thrombocytopenia after ChAdOx1 nCoV-19 vaccination. Lancet.

[46] Panovska-Stavridis I, Pivkova-Veljanovska A, Trajkova S, Lazarevska M, Grozdanova A, Filipche (2021). V. A rare case of superior ophthalmic vein thrombosis and thrombocytopenia following ChAdOx1 nCoV-19 vaccine against SARS-CoV-19. Mediterr J Hematol Infect Dis.

[47] Bialasiewicz AA, Farah-Diab MS, Mebarki HT (2021). Central retinal vein occlusion occurring immediately after 2nd dose of mRNA SARS-CoV-2 vaccine. Int Ophthalmol.

[48] Endo B, Bahamon S, Martínez-Pulgarín D (2021). Central retinal vein occlusion after mRNA SARS-CoV-2 vaccination: a case report. Indian J Ophthalmol.

[49] Bohler AD, Strom ME, Sandvig KU, Moe MC, Jorstad OK (2021) Acute macular neuroretinopathy following COVID-19 vaccination. Eye 1–2. 10.1038/s41433-021-01610-1 10.1038/s41433-021-01610-1 PMC8217204 34158652

[50] Michel TSN, Gascon P, Dupessey F, Comet A, Attia R, David T et al (2021) Acute macular neuroretinopathy after COVID-19 vaccine. 10.21203/rs.3.rs-632137/v1. J Ophthal Inflamm Infect 10.1016/j.jfo.2022.01.022 PMC9130638 35717218

[51] Book BAJ, Schmidt B, Foerster AMH (2021). Bilateral acute macular neuroretinopathy after vaccination against SARS-CoV-2. JAMA Ophthalmol.

[52] Mambretti M, Huemer J, Torregrossa G, Ullrich M, Findl O, Casalino G (2021) Acute macular neuroretinopathy following coronavirus disease 2019 vaccination. Ocul Immunol Inflamm 1–4. 10.1080/09273948.2021.1946567 10.1080/09273948.2021.1946567 34187278

[53] Sanjay S, Gadde SGK, Kumar Yadav N, Kawali A, Gupta A, Shetty R, Mahendradas P “Bilateral Sequential Acute Macular Neuroretinopathy in an Asian Indian Female with β Thalassemia Trait following (Corona Virus Disease) COVID-19 Vaccination and Probable Recent COVID Infection- Multimodal Imaging Study.“. Ocul Immunol Inflamm 2022 Jan 20:1–6. doi: 10.1080/09273948.2022.2026978 10.1080/09273948.2022.2026978 35050826

[54] Singh RB, Singh Parmar UP, Kahale F, Agarwal A, Tsui E (2022) Vaccine-associated uveitis following SARS-CoV-2 vaccination: A CDC-VAERS database analysis. Ophthalmology. Aug 30:S0161-6420(22)00672-8. doi: 10.1016/j.ophtha.2022.08.027. Epub ahead of print. PMID: 36055601; PMCID: PMC9428109

