# Supplementary material for: Ocular manifestations following COVID-19 vaccination
Source: J Ophthalmic Inflamm Infect. 2023 Sep 23;13:44. doi: 10.1186/s12348-023-00358-x (PMC10516807; doi:10.1186/s12348-023-00358-x)
Supplement: Supplementary file 1 — Additional file 1: Graph 1. Percentage of population affected (in weeks) following a dose of COVID-19 vaccination. Graph 2. Type of occurrence of inflammatory manifestations following dose of vaccine. Graph 3. Etiological diagnosis and type of occurrence showing that cases with autoimmune uveitis primarily showed recurrence of inflammation and rarely presented with a first episode of disease [file 12348_2023_358_MOESM1_ESM.docx]

Graph 1. Percentage of population affected (in weeks) following a dose of COVID-19 vaccination.

Graph 2. Type of occurrence of inflammatory manifestations following dose of vaccine.

Graph 3. Etiological diagnosis and type of occurrence showing that cases with autoimmune uveitis primarily showed recurrence of inflammation and rarely presented with a first episode of disease.
